# Triple Reporter Assay: A Non-Overlapping Luciferase Assay for the Measurement of Complex Macromolecular Regulation in Cancer Cells Using a New Mushroom Luciferase–Luciferin Pair

**DOI:** 10.3390/s23177313

**Published:** 2023-08-22

**Authors:** Aaiyas Mujawar, Pratham Phadte, Ksenia A. Palkina, Nadezhda M. Markina, Ameena Mohammad, Bhushan L. Thakur, Karen S. Sarkisyan, Anastasia V. Balakireva, Pritha Ray, Ilia Yamplosky, Abhijit De

**Affiliations:** 1 Molecular Functional Imaging Laboratory, Advanced Centre for Treatment, Research and Education in Cancer, Navi Mumbai 410210, India; amujawar@actrec.gov.in (A.M.); ami.cph.ami@gmail.com (A.M.); 2 Faculty of Life Science, Homi Bhabha National Institute, Mumbai 400094, India; pphadte@actrec.gov.in (P.P.); bhushan0331@gmail.com (B.L.T.); pray@actrec.gov.in (P.R.); 3 Imaging Cell Signalling and Therapeutics Laboratory, Advanced Centre for Treatment, Research and Education in Cancer, Navi Mumbai 410210, India; 4 Institute of Bioorganic Chemistry (IBCh), Russian Academy of Sciences, Moscow 119991, Russia; kpalkina93@gmail.com (K.A.P.); markina.nadya@gmail.com (N.M.M.); karen.s.sarkisyan@gmail.com (K.S.S.); balakireva.anastacia@gmail.com (A.V.B.); 5 Planta LLC, Bolshoi Boulevard, 42 Street 1, Moscow 121205, Russia; 6 Synthetic Biology Group, MRC London Institute of Medical Sciences, London W12 0NN, UK

**Keywords:** bioluminescence, fungal luciferase, triple luciferase assay, gene expression, transcriptional regulation, cancer

## Abstract

This study demonstrates the development of a humanized luciferase imaging reporter based on a recently discovered mushroom luciferase (*Luz*) from *Neonothopanus nambi.* In vitro and in vivo assessments showed that human-codon-optimized *Luz* (*hLuz*) has significantly higher activity than native *Luz* in various cancer cell types. The potential of *hLuz* in non-invasive bioluminescence imaging was demonstrated by human tumor xenografts subcutaneously and by the orthotopic lungs xenograft in immunocompromised mice. *Luz* enzyme or its unique 3OH-hispidin substrate was found to be non-cross-reacting with commonly used luciferase reporters such as Firefly (FLuc2), *Renilla* (RLuc), or nano-luciferase (NLuc). Based on this feature, a non-overlapping, multiplex luciferase assay using *hLuz* was envisioned to surpass the limitation of dual reporter assay. Multiplex reporter functionality was demonstrated by designing a new sensor construct to measure the NF-κB transcriptional activity using *hLuz* and utilized in conjunction with two available constructs, p53-NLuc and PIK3CA promoter-FLuc2. By expressing these constructs in the A2780 cell line, we unveiled a complex macromolecular regulation of high relevance in ovarian cancer. The assays performed elucidated the direct regulatory action of p53 or NF-κB on the PIK3CA promoter. However, only the multiplexed assessment revealed further complexities as stabilized p53 expression attenuates NF-κB transcriptional activity and thereby indirectly influences its regulation on the PIK3CA gene. Thus, this study suggests the importance of live cell multiplexed measurement of gene regulatory function using more than two luciferases to address more realistic situations in disease biology.

## 1. Introduction

Bioluminescence (BL) is an active light-producing event where specific enzyme-substrate catalytic reactions reported in diverse living organisms in nature result in photonic emission. Various BL systems are extensively used as ‘reporters’ in biology experiments for biotechnology and biomedical research applications. Reporter genes are also extensively used for performing non-invasive imaging procedures in various vertebrate, invertebrate, and plant model systems [1]. The advantages of using bioluminescence reporters primarily rely on their photonic emission property via a catabolic process of a specific substrate, thus providing an ultralow background signal. Therefore, BL reporters are generally valued for their sensitivity and specificity marks, providing excellent spatial resolution and signal quantitation [2]. These features of bioluminescence are consequently considered in developing non-invasive bioluminescence imaging (BLI) for real-time visualization of biological functions in vivo occurring in the physiological milieu of live animal models. 

Although the BL phenomenon is widespread, a majority of luminous species are marine inhabitants. Since the time aequorin from *Hydromedusa aequorea* was isolated [3], many new luciferase systems have been discovered. Currently, the most widely used bioluminescence systems are Firefly luciferase (FLuc), *Renilla* luciferase (RLuc), *Gaussia* luciferase (GLuc), and *Oplophorus* luciferase (NLuc) [4,5,6,7,8]. Improved RLuc versions have been used for in vivo tracking in potential stem cell applications [9]. The circularly permutated GLuc, RLuc, and NLuc were also used for the development of protein complementation-based assays [10]. It is also worth noting some of the recent development of luciferase-based sensors for in vivo imaging, cell tracking, and biochemical assays [11,12,13]. Luciferases have been used to detect various inhibitors and bioactive products by using handheld devices as well [14]. In particular, NLuc has been used as a sensor for detecting specific DNA or RNA sequences which can be used as a diagnostic tool for disease biomarkers detection [15]. Paper-based NLuc sensors have been developed to detect toxic chemicals in the air with the help of a smartphone [16]. Alongside using available BL reporters in biological assays and imaging, demand for developing multiplexed options has grown. The importance of multiplexed BL assays to study simultaneous biological events from living systems lies in the undeniable potential to improve assessment reproducibility while minimizing the required number of replicates. However, the upbringing of a truly multiplexed luciferase reporter assay remained constricted as the common marine luciferases in use, i.e., RLuc, GLuc, and NLuc, are all reactive to the coelenterazine or its chemical isoforms as substrate [2,7]. Therefore, technically, all of them can be utilized to measure only one event at a time. For several decades FLuc and RLuc reporter combination has been serving the purpose of gene function assessment as a commercial assay system called ‘dual luciferase assay’ [17]. In this system, one BL reporter can measure the gene regulatory function, while the other BL reporter is used as an internal control for assay normalization [18]. Thus, until now, limited resources of non-cross-reacting Luciferase:Luciferin pairs kept the scope of multiplexed BL reporter-based assessment implausible.

To circumvent this issue, recently, a few studies have attempted to use the spectral unmixing method [19,20,21]. By choosing a combination of three luciferases or their mutational variant with at least a 30–40 nm gap in their emission peaks, the measurements of linked gene activities were shown by quantifying spectral photonic emission. However, unlike fluorescence emission, a broad spectral distribution pattern for any known BL emission causes significant signal overlap. Thus, for any such multiplexed combination, the determination of correction factors during output measurements complicates the process. Therefore, a truly multiplexed assay development by combining non-cross-reacting luciferases remained an unresolved puzzle to mitigate the high demand in the field. Recently, many bioluminescence mushroom species have been reported which are evolutionarily divergent from other known terrestrial or marine species [22,23,24,25]. The BL gene from the wild mushroom species *Neonothopanus nambi* was recently discovered by identifying the intermediates of the luciferin biosynthetic pathway [22,24]. The reported and structurally studied fungi BL system (Luz) can be developed as a promising tool for biomedical research applications.

During this study, we aimed to study the NF-κB, p53 macromolecules in PIK3CA gene regulation because of their significance in chemoresistant ovarian cancer and limited knowledge about their cross-talk [26,27,28,29,30,31]. NF-κB and p53 signaling are well-characterized and elucidated in cancer biology. External cues modulate these two signalings, and their response can be captured by measuring the expression of transcriptional target genes, such as PIK3CA. Regulation of p53 or NF-κB individually regulating the PIK3CA gene transcription can be studied separately using an available dual luciferase assay system. But in situations where both activated p53 and-NF-κB are present, how that may impact PIK3CA transcription was unknown [32,33,34]. Therefore, taking advantage of the conceptualized ‘triple luciferase assay’ by combining hLuz as a third luciferase reporter, this study contributes to developing a new and enhanced understanding of *PIK3CA* gene regulation biology in live cells. 

## 2. Materials and Methods

### 2.1. Materials

Major chemicals and kits used in this study are in Supplementary Table S1. 

### 2.2. Plasmids and Cloning

The vector control pcDNA3.1-CMV or pcDNA3.1-CMV-Luz plasmid were ransformed in *E. coli* DH5α and grown overnight on ampicillin-added LB agar plate. Later single colony was inoculated in LB broth and grown overnight at 37 °C in a shaker incubator to use for substrate cross-reaction experiment in bacteria. Mammalian expression plasmid vectors available to us were used for cancer cell experiments, i.e., *PIK3CA* promoter driven expression of a firefly luciferase2-tandem-dimer tomato fusion reporter (PIK3CA-FLuc2-tdT) [33], pNF-κB-hRL-eGFP [35], p53-NLuc fusion reporter (a gift from Promega, Madison, WI, USA) [36], pcDNA3.1-CMV-Luz, and pcDNA3.1-CMV-β-galactosidase. pcDNA3.1-CMV-hLuz plasmid was synthesized using GeneArt^TM^ (ThermoFisher) service. To prepare the NF-κB-response-element-driven hLuz (pNF-κB-hLuz) plasmid, a PCR-amplified hLuz reporter cDNA was subcloned in between 5′NheI and 3′MluI restriction enzyme sites downstream of the NF-κB response element in the vector. The NF-κB response element contains a 160 bp sequence composed of four tandem copies of the NF-κB consensus binding sequence, followed by a minimal TA promoter [35]. The primers used for hLuz gene amplification are in Supplementary Table S2. 

### 2.3. Cell Lines, Cell Culture and Transfection

As per the supplier’s recommendation, various human cell lines, i.e., HT1080, A2780, and SKOV3, were cultured in DMEM, and the A549 and MCF7 were cultured in RPMI1640 media, supplemented with 10% fetal bovine serum and 1% penicillin-streptomycin solution. Transfection was carried out using Lipofectamine 2000 kit as per manufacturer protocol. For luciferase reporter activity comparison, the HT1080 and A549 cell line was transfected with an equimolar concentration of pcDNA3.1-CMV-Luz, pcDNA3.1-CMV-FLuc2, pcDNA3.1-CMV-hLuz, pcDNA3.1-CMV-hRLuc, pcDNA3.1-CMV-RLuc8.6, pcDNA3.1-CMV-NLuc, and transfection normalization was carried out by using pcDNA3.1-CMV-β-galactosidase plasmid added at 1:10 ratio where applicable. The luciferase activities were measured against respective luciferase substrates, and β-galactosidase activity was measured using Galacto-light plus^TM^ for transfection normalization. SKOV3 cell line transiently transfected with pNF-κB-hLuz and pPIK3CA-FLuc2-tdT constructs was denoted as SKOV3 NP, and when transfected with pNF-κB-hLuz, pPIK3CA-FLuc2-tdT, and p53NanoLuc fusion plasmid was denoted as SKOV3 NPp.

A549 cell lines with stable over-expression of pcDNA3.1-CMV-Luz or pcDNA3.1-CMV-hLuz plasmids were established by Zeocin antibiotic selection over 3 weeks, and the clonal cells with the highest BL emission were propagated for further use. A series of A2780 clones stably expressing pNF-κB-hLuz, pPIK3CA-FLuc2-tdT, or p53NanoLuc fusion plasmid was also established by using zeocin, G418, and puromycin selection, respectively. A2780 cell line stably expressing pNF-κB-hLuz (A2780-NhLuz) was also generated by clonal selection and denoted as A2780-NhLuz. This line was further used to generate cell lines with stable co-expression of pPIK3CA-FLuc2-tdT (indicated as A2780NP) as well as A2780 cell line stably co-expressing pNF-κB-hLuz, pPIK3CA-FLuc2-tdT and p53-NLuc fusion protein (denoted as A2780NPp). A2780 cell lines stably expressing either pPIK3CA-FLuc2-tdT (denoted as A2780pPIK3CA) or p53-NLuc (denoted as A2780PNLuc) were also generated for use. All cell culture experiments were performed at 37 °C with 5% CO_2_ in a humidified incubator.

### 2.4. Protein Sample Preparation and Immunoblotting

Whole-cell extracts were prepared using RIPA buffer with sodium vanadate, sodium fluoride, and a proteinase inhibitor cocktail. After incubation in ice for 30 min, lysates were sonicated and collected after centrifugation at 13,000 rpm for 20 min. For immunoblotting, 40 μg of protein were resolved by SDS-PAGE, transferred onto BioTrace PVDF membrane, and probed using appropriate dilutions of the following primary antibodies as listed in Supplementary Table S3. Detection was carried out by respective HRP-conjugated anti-mouse or anti-rabbit secondary antibodies. Immunoreactive protein complexes were visualized by using the WesternBright ECL kit. Image acquisition and analysis were made on Chemi-Doc imaging systems (Bio-rad).

### 2.5. Quantitative RT-PCR

cDNA was synthesized using 2 μg of total mRNA extracted using an RNA extraction kit from cells as per the manufacturer’s protocol. The qRT-PCR was performed using the SYBR-Green per the manufacturer’s instruction. The relative expression levels of mRNAs were calculated following the standard 2^−ΔCt^ method with GAPDH as an internal control [37]. Primer sequences used are listed in Supplementary Table S2.

### 2.6. Live Cell Imaging

HT1080, A549, MCF7, A2780, or SKOV3 cells (10,000/well) were seeded in 96-well black plates for live cell imaging. These cells were either left untreated or treated with TNFα or doxorubicin or both for specified concentrations and time, as indicated in the results. The BLI signal output from NLuc was captured by adding furimazine (1:1000 dilution), FLuc2 by adding d-luciferin (1 mg/mL), RLuc8.6 by adding coelenterazine h (10 μg/mL) or of Luz/hLuz by adding 3-OH hispidin (1 mg/mL). In some experiments, a variable amount of 3-OH hispidin substrate was used as specified for measuring hLuz activity. Region of Interest (ROIs) was drawn over each well after images were captured, and average radiance (photons/s/cm^2^/sr) was quantified by using the Living Image (version 4.5.4) software.

### 2.7. Bioluminescence Imaging (BLI) of Tumor Xenografts

Animal care and euthanasia were performed per the guidelines and approval of the Institutional Animal Ethics Committee (IAEC) recommendations. For in vivo non-invasive imaging, 5 × 10^6^ A549 lung adenocarcinoma cells over-expressing Luz were injected subcutaneously at the right fore-limb position, and the same amount of A549 hLuz over-expressing cells at the right hind-limb position in nude mice (*n* = 3). For imaging from deep tissue organs, 1 × 10^6^ cells of A549hLuz or Luz cell line suspended in sterile 1× PBS (pH 7.4) were injected intravenously via tail vein in two sets of nude mice *(n* = 3). BLI was performed 30 min after cell implantation, and sequential image acquisition was carried out between 5–15 min after intraperitoneal injection of 3OH-hispidin (3 mg in 100 μL sterile PBS). For the tumor growth study, Nod-SCID mice *(n* = 4) were subcutaneously implanted with 5 × 10^6^ cells of A549 hLuz over-expressing cells at the hind flank location. Substrate injection and BLI image acquisition protocol were the same as before using the IVIS Lumina II imaging system. ROIs were drawn over the tumor signal, and average radiance (photons/s/cm^2^/sr) was quantified using the LivingImage (version 4.5.4) software.

### 2.8. Chromatin Immunoprecipitation

The ChIP experiment was performed as described earlier with few modifications [36,38]. In brief, 1 × 10^7^ cells of the baseline and p53 overexpressing A2780 ovarian cancer cell line (A2780PNLuc) were treated with doxorubicin (1 μg/mL), TNFα (10 ng/mL), and a combination of doxorubicin + TNFα for 24 h. Untreated cells were kept as a control and cross-linked by using 0.75% formaldehyde. Nuclei collected in CHIP lysis buffer (50 mM HEPES-KOH pH7.5, 140 mM NaCl, 1 mM EDTA pH8, 1% TritonX 100, 0.1% SDS, 0.1% Sodium Deoxycholate, and proteinase inhibitor) were sonicated to obtain an average of 200–400 bp chromatin fragments. A total of 25 μg of chromatin was precipitated with either anti-p53 or anti- NF-κB p65 antibody, and the DNA was eluted after reverse–cross-linking. Non-immunoprecipitated chromatin was used as total input control. Further, ChIP-DNA was analyzed with real-time PCR using site-specific primers (Supplementary Table S2), and the fold enrichment was determined.

### 2.9. Statistical Analysis

All in vitro and in vivo data comparisons were conducted using Welch’s modification Student’s *t*-test by using GraphPad prism unless otherwise stated. Error bars represent SEM from at least three independent experiments. Values of *p* < 0.05 were considered statistically significant.

## 3. Results

### 3.1. Human Codon Optimization Improved Wild-Type Mushroom Luciferase (Luz) Reporter Activity

**Figure 1:**
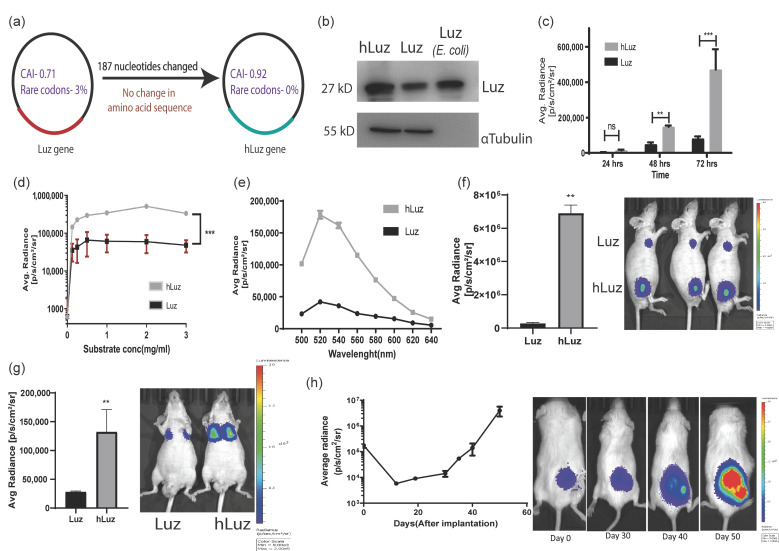
Humanized Luz as an optical reporter for in vitro and in vivo imaging. (**a**) Schematic diagram highlighting main parameters of human codon optimization process applied on wild-type Luz gene. (**b**) Immunoblot of hLuz and Luz expression in HT1080 cell line (purified Luz protein from *E. coli* was used as an independent control). (**c**) Comparison of Luz and hLuz reporter activity at 24, 48, and 72 h after transfection of the respective plasmids in live HT1080 cells. (**d**) The activity of Luz and hLuz with respect to increasing substrate concentration in live HT1080 cells. (**e**) Comparison of Luz and hLuz protein emission spectra measured from live HT1080 cells added with the 3OH-Hispidin substrate. (**f**) Bar graph and BLI image comparing luminescence photon output of A549 cell line expressing either Luz or hLuz cells implanted in nude mice. (**g**) Bar graph and BLI image comparing Luz and hLuz photon output after orthotopic implantation of one million A549-hLuz or A549-Luz cells by intravenous tail injection in nude mice. (**h**) Line graph and BLI images showing A549-hLuz xenograft growth in subcutaneous location over 50 days. Mean ± SEM plotted, ns—non-significant **—*p* = 0.01 ***—*p* = 0.001.

When the wild-type *N. nambi* gene was expressed in a mammalian cell, the Luz protein showed weak luciferase activity in the presence of its 3OH-hispidin substrate. We predicted that a reason for a weak luciferase signal might be the non-human codon bias in fungal expression systems affecting Luz expression and mRNA stability, leading to lower expression of Luz protein [39,40,41]. Therefore, to rule out the codon bias and improve the scope of this fungal luciferase in biomedical applications as an independent reporter gene, we decided to perform human codon optimization of the native Luz gene. A thorough analysis of the cDNA sequence was performed for human cell expression using the GeneOptimizer^TM^ web tool [42]. Results showed that the Luz gene has a codon adaptive index (CAI) of 0.71, with 52.15 GC content and 3% rare codons (Table 1). The lower CAI index and the presence of a significant number of rare codons affected the native Luz expression, thus impacting its overall expression in the human cell system. Thereafter, the human-codon-optimized Luz (hLuz) gene was synthesized by replacing 187 nucleotides in the Luz gene (Supplementary Figure S1a). The absence of rare codons in the hLuz DNA sequence was reconfirmed using the GeneOptimizer^TM^ toolbox [42]. The CAI index value improved significantly to 0.92, along with 56.85% GC content (Figure 1a). It is also worth noting that nucleotide sequence comparison between hLuz and Luz using Needleman–Wunsch global alignment webtool showed a 78% identity match with no change in amino acid sequence (Table 2) [43].

Following this development, Luz and hLuz expression plasmids were transfected in the HT1080 cancer cell line, and their protein expressions were verified by immunoblotting using LUZ polyclonal antibody. Semi-quantitative immunoblotting indicated that the hLuz plasmid produced a much higher amount of protein than the Luz-expressing plasmid, despite being under the same promoter control. As an independent size reference, 20 μg of purified LUZ was loaded in the same gel (Figure 1b). Luciferase activity was measured from the HT1080 cell line transfected with an equivalent amount of Luz or hLuz plasmid DNA, in which hLuz showed 5-fold higher photon output at 24 h time-point and 5.8-fold higher at 72 h (*p* = 0.0004) than Luz (Figure 1c). The photon output of hLuz was at least 8-fold higher than native Luz in the MCF7 cancer cell line (*p* = 0.0006) (Supplementary Figure S2). At different concentrations of 3OH-hispidin substrate, hLuz and Luz showed maximum photon output at 0.5–1 mg/mL substrate concentration (Figure 1d). Therefore, from now onwards, 1 mg/mL 3-OH hispidin was used as the standard concentration for all in vitro experiments. Further, the emission spectra of Luz and hLuz were compared and revealed no significant change in emission maxima (Figure 1e). Therefore, humanized Luz showing substantial improvement in its bioluminescent activity while maintaining the spectral properties is likely because of the improved mRNA stability and expression in human cells. 

Later A549 clonal cell lines with stable overexpression of Luz (A549-Luz) and hLuz (A549-hLuz) were established. The photon output from an equal number of A549-Luz and A549-hLuz cells were compared in vivo by implanting these cells subcutaneously in the nude mice strain. As expected hLuz cells showed 6.894 × 10^6^ photon/s/cm^2^/sr, which was ~25-fold higher than Luz (2.746 × 10^5^ photon/s/cm^2^/sr), whereas the background signal was only 2666 photon/s/cm2/sr (Figure 1f). Further, by delivering one million A549-hLuz or A549-Luz cells via tail-vein in two sets of mice, potential improvement as a BLI reporter for deep tissue imaging ability was judged. Specific cell localization at the orthotopic site, i.e., lungs, was observed with A549-Luz and A549-hLuz cells. The photon output for hLuz was 32,615 photon/s/cm^2^/sr, which was 3 times higher than Luz (11,106 photon/s/cm^2^/sr), whereas the average background signal was 1413 photon/s/cm2/sr (Figure 1g).

The utility of hLuz as an imaging reporter to monitor A549-hLuz cancer cell growth in a preclinical mouse model was verified over seven weeks. It was found that an average radiance of 4.44 × 10^5^ photon/s/cm^2^/sr at the subcutaneous tumor site on the day of implantation, which was well above the average background signal of 1.59 × 10^3^ photon/s/cm^2^/sr (Figure 1h). As the tumors grew, the BLI signal reciprocally increased to an average of 2.041 × 10^6^ photon/s/cm^2^/sr on day 50. These results confirmed that with improved photon output, hLuz might be used as a better imaging reporter as compared to native Luz in preclinical experiments.

### 3.2. Luz and 3OH-Hispidin Combination Is a Novel Non-Cross-Reacting Bioluminescence Reporter System

**Figure 2:**
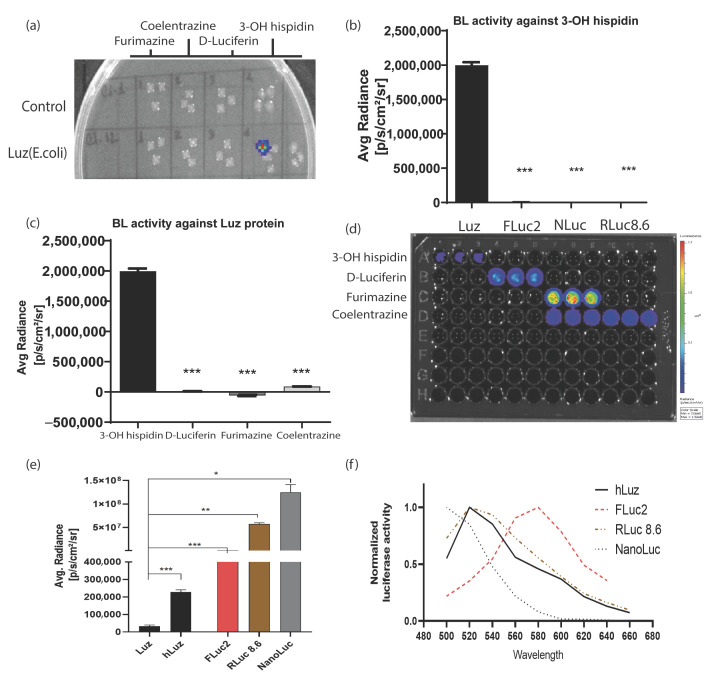
Luz reporters do not cross-react with other commonly used luciferases. (**a**) Image output of a bacto–agar plate with live control and Luz–expressing E. coli colonies grown overnight to test cross-reactivity against various luciferase substrates. (**b**) Bar graph representing average max photonic emission computed from HT1080 live cells over-expressing Luz, FLuc2, NanoLuc, and RLuc8.6 in the presence of 3–OH hispidin substrate only. (**c**) Bar graph representing average max photonic emission computed from Luz-expressing cells only in the presence of 3–OH hispidin, D–luciferin, Furimazine, and coelenterazine substrates. (**d**) Image of the 96–well black plate from which the graphs (**b**,**c**) were analyzed. (**e**) Bar graph showing the comparative photon output of Luz, hLuz, and three other non–overlapping common luciferases (i.e., FLuc2, RLuc8.6, and NanoLuc) with their respective substrates. The photonic signal was measured 24 h after transfection of the respective expression plasmids in the HT1080 cell line, and mean ± SEM plotted for triplicate samples; ns—non–significant *—*p* = 0.05, **—*p* = 0.01 ***—*p* = 0.001. (**f**) The line chart showing the spectral pattern of hLuz, FLuc2, RLuc8.6, and NanoLuc captured using an IVIS spectrum imager with 20 nm bandpass filters covering the 500–660 nm spectral range.

To check the possibility of developing a multiplexed BL assay using an hLuz reporter, several tests were performed to understand potential cross-reactivity with other commonly used BL luciferase and their substrates. As expected, the bacterial colonies expressing Luz-deficient control plasmid did not yield any BL signal for any of the four substrates tested, whereas the colonies expressing Luz plasmid showed the BL signal only in the column added with 3-OH hispidin substrate (Figure 2a). To further elaborate on the cross-reactivity aspect of Luz, we chose well-established mammalian expression plasmids of FLuc2 [44], RLuc8.6 (a brighter and red-shifted mutant variant of native *Renilla* luciferase) [45], and NanoLuc reporters [46]. An equivalent amount of plasmid DNAs of Rluc8.6, Fluc2, and NanoLuc were transfected in parallel along with hLuz plasmid in the HT1080 cancer cells and tested individually by adding their respective substrates. As this result suggests, only the Luz-expressing cells showed luciferase activity in the presence of 3-OH hispidin and not with any other substrates (Figure 2b,d). Additionally, no luminescence photon output was obtained from the other three luciferase cell-containing wells added with 3-OH hispidin substrate (Figure 2c,d).

Thereafter, the luciferase reporter activity of Luz or hLuz was compared with FLuc2, RLuc8.6, and NanoLuc against their respective substrates using two cancer cell lines. The result showed that in the HT1080 cell, the average photon output from both Luz or hLuz (32,115 ± 6468 photon/s/cm^2^/sr and 227,998 ± 10,151 photon/s/cm^2^/sr, respectively) were lower than the rest of the other three luciferases tested (Figure 2e). In the A549 cell, compared to Luz, all three commonly used luciferases showed significantly higher photon yield (*p* > 0.05) as well (Supplementary Figure S3). Further, the emission spectrum of hLuz was compared with the other luciferase reporters, where it was observed that the hLuz emission spectrum closely overlapped with RLuc8.6, which is a red-shifted RLuc variant [45], but was widely separated from NanoLuc and FLuc2 reporters as their Em^Max^ are known to be 460 nm [46] and 610 nm [44], respectively (Figure 2f). Therefore, considering all these parameters, we selected FLuc2 and NanoLuc reporters to perform a non-overlapping and spectrally resolvable multiplexed luciferase assay in combination with the hLuz reporter. 

### 3.3. NF-κB Regulatory Response Can Be Measured by Using hLuz Reporter Sensor

**Figure 3:**
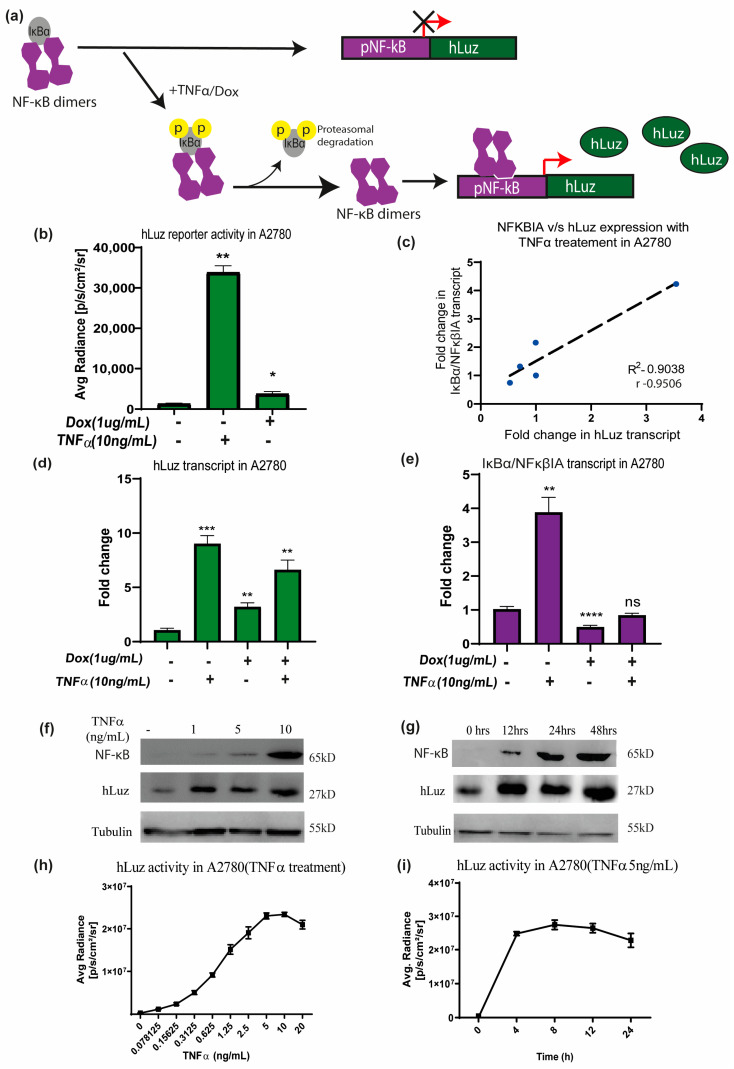
The hLuz reporter expression to measure NF-κB activation. (**a**) Schematic overview of NF-κB reporter sensor function to measure transcriptional regulatory activity using hLuz. (**b**) Effect of TNFα (10 ng/mL) and doxorubicin (1 μg/mL) treatment for 24 h on hLuz activity in A2780 cell line transiently transfected with pNF-κB-hLuz plasmid. (**c**) Line graph showing linear correlation of hLuz and NFκBIA transcript expression in A2780-NhLuz cell line treated with variable TNFα concentration (0.3125, 0.625, 1.25, and 2.5 ng/mL) for 24 h. (**d**) Bar graph showing hLuz transcript and (**e**) NFκBIA transcript expression in A2780-NhLuz cell in untreated or treated condition with 10 ng/mL TNFα, 1 μg/mL doxorubicin, and both for 24 h. (**f**) Immunoblot showing NF-κB (RelA) and hLuz expression in the A2780-NhLuz cell line treated with different concentrations of TNFα for 24 h. (**g**) Immunoblot showing NF-κB (RelA) and hLuz expression in A2780-NhLuz cell line at different time points after treatment with TNFα (10 ng/mL). (**h**) Concentration-dependent effect on hLuz activity in A2780-NhLuz cell line treated with TNFα for 24 h. (**i**) Time-dependent effect on hLuz activity in A2780-NhLuz treated with TNFα (5 ng/mL). (Mean±/− SEM plotted for n = 3, ns—non-significant, *—*p* = 0.05, **—*p* = 0.01 ***—*p* = 0.001 ****—*p* = 0.0001).

To use hLuz as an optical reporter sensor for NF-κB transcriptional activation, the cDNA of hLuz was subcloned in a plasmid vector downstream of the NF-κB response element regulatory sequence (Supplementary Figure S1b). When the cellular condition allows NFκBIA (or IKB) to dissociate (a well–established NF-κB target gene [47]) and resulting free NF-κB to translocate to the nucleus and bind at the NF-κB response element, it will drive the hLuz expression and a signal gain will be observed (Figure 3a). Thereafter, this sensor DNA was used for establishing A2780 ovarian cancer cell line over–expressing pNF-κBhLuz plasmid (A2780-NhLuz cell line). Both hLuz and NFκBIA transcripts were measured by real–time PCR in the presence or absence of TNFα and doxorubicin modulators [48,49]. Compared to the untreated cells, treatment with TNFα for 24 h showed a 14–fold increment (*p* = 0.0001) of hLuz transcript, while NFκBIA transcript showed a 5–fold increase (*p* = 0.0066). For doxorubicin treatment, the expression of hLuz increased significantly (*p* = 0.0026), but NFκBIA (*p* = 0.0001) expression decreased significantly. Combined treatment of doxorubicin and TNFα showed increased hLuz transcript expression (*p* = 0.0012) and non–significant change in NFκβIA transcript (Figure 3d,e). The co–relation between NFκBIA and hLuz transcript expression in the A2780-NhLuz cell line treated with varying concentrations of TNFα was also checked and found a linear correlation value of 0.95 with a significance of *p* = 0.013 between the expression of hLuz and NFκβIA (Figure 3c and Supplementary Figure S4). Further, in A2780-NhLuz cells compared to untreated condition, measured luciferase activity showed several folds of increment when treated with TNFα (*p* = 0.0025) and about 2–fold increment when treated with doxorubicin (*p* = 0.046) (Figure 3b).

Further, immunoblotting was performed for semi–quantitative estimation of NF-κB (RelA) and hLuz proteins in the A2780-NhLuz cell. Compared to the untreated condition, treatment with 1, 5, and 10 ng/mL of TNFα showed a profound increase in hLuz protein and a concentration–dependent increment of NF-κB protein (Figure 3f). Further, the A2780-NhLuz cells treated with TNFα (10 ng/mL) showed a significant increase of hLuz protein in samples incubated for 12 h, 24 h, and 48 h compared to untreated samples. NF-κB (RelA) expression showed a trend of increment with increasing treatment TNFα time (Figure 3g). Thereafter, the A2780-NhLuz cell line was treated with increasing concentrations of TNFα ranging from 0 to 10 ng/mL) and found increased hLuz activity starting from as low as 0.15 ng/mL of TNFα treatment (Figure 3h). For further characterization, time-dependent kinetics of hLuz activity were also measured, and a time-dependent increase in the activity post-TNFα treatment (5 ng/mL), which reached near saturation within 8 h of treatment (Figure 3i), was observed. Thus, hLuz as a reporter, can measure both concentration and time-dependent effects of TNFα on NF-κB transcriptional activity.

Thereafter, the effects of the same modulators were tested in A2780pPIK3CA cells over-expressing the PIK3CA promoter driving FLuc2-tdT fusion reporter. As expected, the A2780pPIK3CA cell line treated with TNFα showed a 3–fold increase in PIK3CA promoter activity (*p* < 0.0001) and a significantly decreased activity with doxorubicin treatment (*p* = 0.0076) (Supplementary Figure S5). Further, to bring in a third luciferase in the context of developing a triple luciferase system, a p53-NLuc fusion plasmid construct was used, where modulated NLuc signal was tested to indicate p53 activation and stabilization. So, in the A2780 cells with p53-NLuc over-expression (A2780PNLuc) when treated with doxorubicin, a highly significant increase in NLuc activity was observed as expected compared to untreated cells (Supplementary Figure S6). 

### 3.4. Application of Triple Luciferase Assay Revealed Complex Regulation of PIK3CA Gene Promoter by p53 and NF-κB

**Figure 4:**
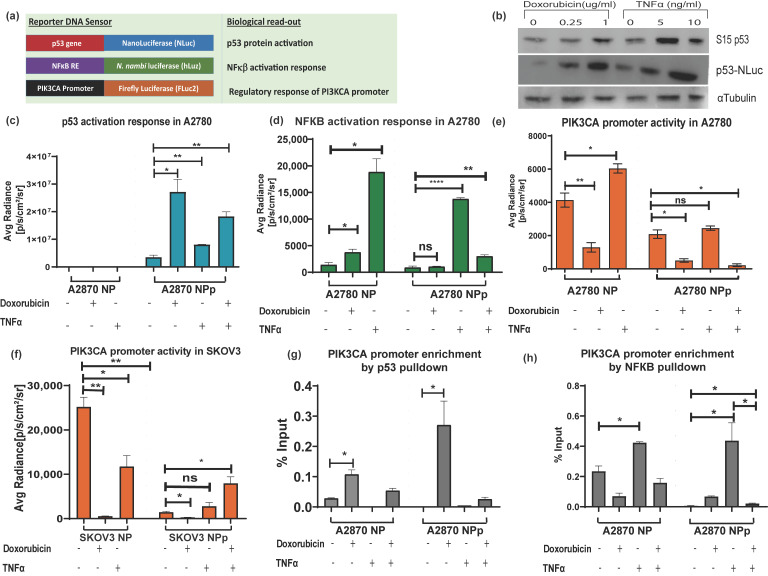
Demonstration of triple luciferase reporter assay to study complex gene regulation mechanism. (**a**) Schematic details of the reporter constructs combined to measure NF-κB regulation activity, p53 activation response, and PIK3CA promoter activity. (**b**) Immunoblot analysis of S15 phosphorylated p53 and p53-NLuc expression measured in the A2780NPp cell line before and after treatment with doxorubicin and TNFα for 24 h. Effect of TNFα (10 ng/mL), doxorubicin (1 μg/mL), and p53 overexpression in A2780NPp cell line on (**c**) p53 expression, (**d**) NF-κB transcriptional activity, and (**e**) PIK3CA promoter activity. (**f**) Effect of TNFα (10 ng/mL), doxorubicin (1 μg/mL), and p53 overexpression in SKOV3 cell line on PIK3CA promoter activity. (**g**) CHIP assay for p53 binding to PIK3CA promoter represented as fold enrichment. (**h**) CHIP assay for NF-κB binding to PIK3CA promoter represented as fold enrichment. (Mean ± SEM plotted for n = 3, ns—non-significant *—*p* = 0.05, **—*p* = 0.01, ****—*p* = 0.0001. All the groups are compared to the untreated group using Welch’s modification Student’s *t*-test by using GraphPad prism unless otherwise stated).

After establishing the pNF-κB–hLuz sensor, we decided to combine the p53-NLuc and pPIK3CA-FLuc2-tdT sensors to study a complex gene regulation of PIK3CA promoter by p53 and NF-κB (Figure 4a). The three-reporter system provided the opportunity to measure NF-κB activation, p53 activation, and PIK3CA promoter regulation directly or indirectly by estimating hLuz, NLuc, and FLuc2 reporter activity, respectively. It was observed that 24 h treatment with doxorubicin (1 μg/mL) and TNFα (10 ng/mL) or both could activate and thereby stabilize the p53 protein in the A2780 cell line. Compared to untreated cells, expression and stabilization of p53-NLuc fusion protein were evident in immunoblot experiments performed after 24 h treatment of the A2780 cell line with two different concentrations of doxorubicin and TNFα (Figure 4b). Further, compared to untreated conditions, high NLuc photon emission from A2780NPp cells treated with doxorubicin (*p* = 0.017) indicated stabilization of the p53-NLuc fusion protein. Treatment of TNFα alone also showed significant (*p* = 0.007) enhancement in the NLuc signal. Since A2780NP cells lacked p53-NLuc protein, no luciferase output was observed. Interestingly, a combined treatment of doxorubicin and TNFα showed an increment of luciferase signal (*p* = 0.0012); however, it was lesser than in the doxorubicin treatment group, indicating a competitive modulation effect on p53 stabilization (Figure 4c). Additional support came in from similar experiments performed using the SKOV3 cell line, which is reportedly an endogenous p53 null cell line. In the SKOV3NPp cell, a highly significant p53 stabilization effect was evident in the presence of doxorubicin, but this effect was somewhat less pronounced in both doxorubicin and TNFα (Supplementary Figure S7a).

Next, we measured NF-κB activity in the A2780NP cell line, where compared to the untreated cells, hLuz emission indicated a 13–fold increment of NF-κB activity (*p* = 0.01) with 24 h of TNFα (10 ng/mL) treatment. The A2780NP cell line treated with doxorubicin (1 μg/mL) for 24 h showed a 2–fold increment (*p* = 0.035). However, A2780NPp cells treated with TNFα showed a ~10–fold increase of NF-κB activity (*p* = 0.0001) compared to the untreated cells (Figure 4d). The A2780NPp cells treated with doxorubicin showed a non-significant increase in NF-κB activity than untreated cells (*p* = 0.011). Interestingly, A2780NPp cells treated with both TNFα and doxorubicin treatment also recorded a minimal rise in NF-κB activity compared to untreated or only doxorubicin-treated cells. It was also noticed that compared to the A2780NP cell line, the A2780NPp cells in the untreated group showed a non-significant difference in NF-κB transcriptional activity, indicating that basal NF-κB response activity was unaffected by p53 overexpression. However, SKOV3NPp cells showed lesser NF-κB response after treatment with TNFα, doxorubicin, or both than the untreated control condition (Supplementary Figure S7b).

Finally, the effect of modulators on the PIK3CA promoter regulation was measured by FLuc2 reporter activity. A2780NP cell line treated with TNFα showed several folds increase in Fluc2 luciferase activity (*p* = 0.025) compared to the untreated cells. In line with the established fact that p53 negatively regulates PIK3CA promoter [36], we observed a marked decrease in luciferase activity after 24 h of doxorubicin treatment (*p* = 0.007) (Figure 4e). Compared to the A2780NP cell, in the A2780NPp cell line, PIK3CA promoter activity also decreased significantly (*p* = 0.021), and a further dip in PIK3CA promoter was observed in p53 stabilized condition caused by doxorubicin treatment (*p* = 0.009). In the case of combination treatment, A2780NP and A2780NPp cells did not show any significant increase in PIK3CA activity (*p* = 0.047). In the SKOV3 cell line with exogenous p53 (SKOV3-NPp), the PIK3CA promoter activity was also suppressed by p53 activation but enhanced in the presence of TNFα (Figure 4f). These results indicated that TNFα acts as a positive regulator, but its regulatory effect on the PIK3CA promoter is compromised when p53 is over-expressed and stabilized. 

The above results were further validated by performing a ChIP assay to understand the binding ability of p53 and NF-κB on the PIK3CA promoter in A2780 and A2780 p53 overexpressed cell lines treated with doxorubicin, TNFα, or both modulators. Increased physical binding of p53 on the PIK3CA promoter was observed in A2780 cells treated with doxorubicin alone (*p* = 0.012) or with combined doxorubicin and TNFα treatment for 24h (*p* = 0.04) (Figure 4g). In A2780 p53 overexpressed cells, p53 binding to the PIK3CA promoter also increased significantly upon doxorubicin treatment (*p* = 0.44) compared to untreated cells. However, treatment of TNFα alone decreased the binding of p53 on the PIK3CA promoter. 

We found that TNFα treatment increased NF-κB binding to the PIK3CA promoter region in the A2780 cell line. The over-expression of p53 and doxorubicin treatment decreased NF-κB binding to the PIK3CA promoter, and a further decrease was seen with a combination treatment of TNFα and doxorubicin. We observed that the binding of NF-κB to PIK3CA promoter in doxorubicin-treated p53-NLuc overexpressed A2780 cell line was lower than wild-type p53 expressing A2780 cell line (Figure 4h). Further, immunoblotting for p21 in the A2780 cell line treated with either doxorubicin alone or together with TNFα showed increased p21 expression, a downstream target gene of p53 [50,51]. Also, decreased expression of BCL2 when cells were treated with doxorubicin or TNFα and doxorubicin combination indicated a negative impact of p53 stabilization on NF-κB transcriptional activity [52] (Supplementary Figure S8).

## 4. Discussion

The field of biology and biotechnology had to wait for a long time for a breakthrough development to have the choice of the non-overlapping luciferase–luciferin systems currently available, thus enabling the design of multiplexed sensors providing sensitive luminescence readout. The discovery of the mushroom luciferase gene from *N. nambi* and elucidation of its substrate chemistry has provided the opportunity to support experimental studies aimed at understanding complex gene regulation mechanisms in one go. To understand the suitability of this new luciferase reporter for such multiplexed applications, we first tested the Luz reporter enzyme and its unique substrate for cross-reactivity with other major luciferases. We found its unique status among the other commonly used luciferases tested. However, the expression and enzymatic activity of the native Luz in the human cell system was found to be weak. We considered that such weak expression in cancer cells might be due to its lower codon adaptive index and/or the presence of rare codons that are not adequately expressed in the human cell system. Therefore, during this study, the native *Luz* gene was suitably adapted for applications of human cells by performing rational mutagenesis of 187 bases. The humanized *Luz* reporter gene was synthesized, ensuring the amino acid sequence remained intact. After that, the hLuz gene was tested for reporter sensitivity improvements, and in vitro tests performed using multiple human cancer cells showed several folds higher photon output than the native Luz gene. It was also evident that the substrate utilization and the spectral pattern parameters were well maintained in hLuz.

Further, in immunocompromised mice, the hLuz reporter was tested for in vivo BL imaging of A549 lung adenocarcinoma cells implanted at subcutaneous and lung orthotopic locations. The potential of hLuz as a reporter protein for non-invasive imaging to follow tumor growth kinetics in mice models was validated. Together, these results suggested that the Luz reporter system does not overlap with other common luciferases, proving that the humanized mushroom luciferase is a new and evolutionary diverse entity among the BL systems available. Compared to other luciferases in use (i.e., FLuc2, RLuc8.6, and NLuc), the photon output from hLuz is still weaker, which can be enhanced by either substrate modifications or gene mutagenesis in the future. The emission spectrum of Luz is distinct from FLuc2, NLuc, and hRLuc, whereas its spectral maximum overlaps with RLuc8.6 [53]. 

We then approached to design a reporter sensor using the hLuz DNA sequence placed under the regulation of the NF-κB response element. The pNF-κB-hLuz construct was tested for NF-κB regulation activity measurement in A2780 and SKOV3 cell lines treated with external modulators like TNFα, doxorubicin, and p53 overexpression as an internal modulator. Results indicated highly co-relative transcript expression of hLuz and NFκBIA genes. The NFκBIA-encoded protein is a known inhibitor of NF-κB, which interacts with REL dimers to inhibit NF-κB/REL complexes and the well-known NF-κB target gene. Increased transcript expression of both NFκBIA and hLuz in incremental TNFα-treated conditions confirmed that hLuz expression is governed by NF-κB binding ability at the response element site provided. The observed difference in NFκBIA and hLuz transcript expression under doxorubicin treatment might be due to a secondary modulatory effect on NFκBIA caused by non-canonical activation of NF-κB [54]. Further, the TNFα concentration and time-dependent change in hLuz and NF-κB/REL protein expression and reporter activity were measured, indicating the potential use of the sensor. The above results concluded that the pNF-κB-hLuz reporter sensor could capture the cellular response to NF-κB modulations in relevant cancer cells. 

To date, only two non-cross-reacting luciferase–substrate pairs, i.e., FLuc2-D-Luciferin and RLuc–coelenterazine, were known and commonly used in the dual luciferase assay format. Other multiplexed luciferase assays developed were based on substrate modification or mutant luciferases providing specificity with altered emission maxima [21,55,56]. The non-overlapping nature of fungal luciferase in terms of its distinct substrate and emission spectral peak makes it an ideal third luciferase candidate forming the basis for developing a ‘triple luciferase assay’. We combined FLuc2, NLuc, and hLuz in this new assay format to unravel any unknown regulatory insights by simultaneously monitoring the complex gene regulation in epithelial ovarian cancer (EOC). Previous reports suggested that the PIK3CA promoter has binding sites for both p53 and NF-κB [33,34]. Also, in EOC cells, it was shown that p53 acts as a transcriptional repressor of the PIK3CA promoter by directly binding to its promoter sequence [36], whereas in the case of cisplatin-resistant EOC cells, enhanced NF-κB activity is required to develop acquired chemoresistance [14,16]. However, to date, it was unknown whether NF-κB activation may exert indirect control on p53 protein activation and thereby may alter PIK3CA gene regulation. Therefore, we aimed to capture such information on PIK3CA promoter regulation triggered via activated p53 and/or NF-κB modulation. 

Substantial evidence suggests that in response to DNA damage, the p53 protein is activated, and the stabilized p53 thereby forms a complex with p300/CBP. On the other hand, the NF-κB complex is also known to bind to the p300/CBP to carry out its transcriptional activity [57]. Thus, considering the pool of CBP/p300 protein in cells at any given condition is constant, both p53 and NF-κB may have to compete to form complexes with CBP/p300 or one of the component proteins as a pre-requisite to exert their respective transcriptional regulation activity [32,58]. Either the over-expression or stabilization of p53 can make CBP/p300 unavailable for NF-κB binding, resulting in decreased DNA binding and transcriptional regulation of NF-κB [32,58,59]. To analyze such complexity involved in PIK3CA gene regulation by both p53 and NF-κB candidate proteins, we would require at least three independent measures to simultaneously assess the events occurring in cells at any given time. Therefore, by combining the new pNF-κB hLuz sensor with the available luciferase vectors p53-NLuc fusion and pPIK3CA-FLuc2tdT, we designed a multiplexed assessment method to reveal complex regulation of PIK3CA gene expression by NF-κB and p53.

**Figure 5:**
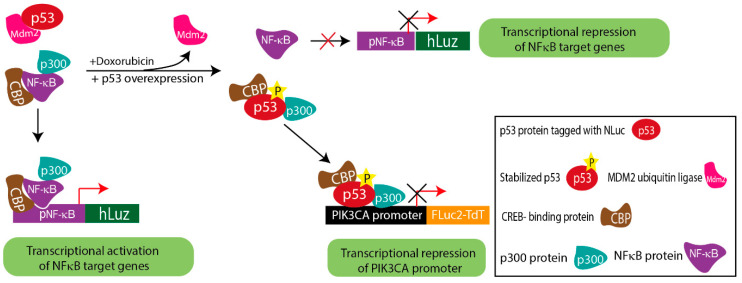
Schematic diagram explaining the complex regulatory mechanism depicted by triple luciferase assay.

The obtained results from the co-expression of the above constructs in A2780 and SKOV3 ovarian cancer cells indicated that stabilized p53 transcriptionally down-regulated PIK3CA and attenuated the transcriptional activity of NF-κB. The ChIP assay supported the conclusion drawn based on the results of the triple luciferase assay. By comparing doxorubicin treated vs. untreated conditions, the ChIP results established that p53 had higher binding potential on the PIK3CA promoter in doxorubicin-treated A2780 cells. Also, in the A2780 cell overexpressing p53, a higher binding of p53 on the PIK3CA promoter than the baseline A2780 cell was observed. This result matched with lower FLuc2 reporter activity, indicating PIK3CA transcriptional repression due to direct binding on the promoter sequence. However, like in the reported case of inflammatory chemoresistant EOC, a more complex situation prevails, where the doxorubicin drug action may counter TNFα action. Due to the lesser binding potential of NF-κB on the PIK3CA promoter, stabilized p53 is a dominant regulator of PIK3CA. Therefore, together these results imply that in a cellular condition with triggered NF-κB, suppressive p53 activation may prevail, which in turn may drive a less suppressive regulatory action of the PIK3CA gene promoter (Figure 5). 

In conclusion, the current study reveals a set of sequential developments required for adopting mushroom luciferase as a unique reporter for non-invasive BL imaging and gene function assessment in live cells. For the first time, we showed that this luciferase protein can be used as a standalone reporter returning sufficient photonic output required for measurement in vitro and in vivo. Second, to ensure optimal performance in biotechnology and biomedical research applications, we prepared a humanized version of the Luz reporter and validated its performance improvements. Third, in conjunction with the established luciferases commonly used, like Fluc2 and NLuc, a multiplexed live cell assay named ‘triple luciferase assay’ was developed, thereby enabling a deeper understanding of complex biological functions of regulatory proteins. Using ovarian cancer cells, triggered regulatory processes were measured via luciferase reporter readout. Further, the assay developed is compatible with the standard set of luminescence measurement equipment available and, therefore, can be widely used for many suitable applications.

We estimate that the current assay system demonstrated here is unique and will support a wide variety of biological estimations in other human diseases. In the future, owing to the unique biochemical mechanism of photonic production, a greater understanding of the hLuz protein structure, guided mutagenesis on its gene sequence, and/or chemical alterations in the 3OH-Hispidin chemical structure will help to expand the scope of use. Adaptation to other multiplexed luciferase assay formats, such as reporter complementation or bioluminescence resonance energy transfer (BRET)-based sensor design, will open up new avenues for this luciferase reporter.

## Figures and Tables

**Table 1:** Comparison of Luz and hLuz gene for optimum protein expression parameters in human cell system analyzed by GeneOptimizer^TM^ web tool.

	Codon Adaptive Index	GC Content	Codon Frequency Distribution
**Standard**	**0.8–1.0**	**30–70%**	**<30%**
**Luz**	**0.71**	**52.15%**	**3%**
**hLuz**	**0.92**	**56.85%**	**0%**

**Table 2:** Nucleotide and amino acid sequence comparison between Luz and hLuz was conducted by Needleman–Wunsch global alignment web tool (Blastn and Blastp, respectively).

Nucleotide sequence alignment of Luz and hLuz				
**Max score**	**Total score**	**Query cover**	**E value**	**Identity**
**627**	**627**	**96%**	**0.0**	**78%**
**Amino acid sequence alignment of Luz and hLuz**				
**Max score**	**Total score**	**Query cover**	**E value**	**Identity**
**554**	**554**	**100%**	**0.0**	**100%**

## Data Availability

The data presented in this study are available on request from the corresponding author.

## Supplementary Materials

The following supporting information can be downloaded at https://www.mdpi.com/article/10.3390/s23177313/s1. Supplementary Table S1: Major chemicals and assay kits used in the study. Supplementary Table S2: Oligo sequences used in the study. Supplementary Table S3: Antibodies used in the study. Supplementary Table S4: Information on Luz and other luciferase used in triple luciferase assay system. Supplementary Figure S1: Plasmid map of (a) pcDNA3.1+hLuz and (b) RE-NFκβ-hLuz expression vectors. Supplementary Figure S2: Bar graph showing Luz and hLuz activity in MCF7 cell line. Supplementary Figure S3: Bar graph showing comparative luciferase activity of Luz with FLuc2, RLuc8.6 and NanoLuc in A549 cell line. Supplementary Figure S4: Line graph showing fold change of hLuz and NFκβIA transcript expression compared to control in A2780-NhLuz cell line treated with variable TNFα concentration (0.3125, 0.625, 1.25 and 2.5 ng/mL) for 24 h. Supplementary Figure S5: Bar graph showing PIK3CA promoter activity in A2780pPIK3CA cell line after treatment of TNFα (10 ng/mL) and Doxorubicin (1 µg/mL) for 24 h. Supplementary Figure S6: Bar graph showing activation of p53 measured by NLuc activity in A2780 cell line over-expressing p53-NLuc fusion protein in presence or absence of Doxorubicin. Supplementary Figure S7: Effect of TNFα (10 ng/mL), Doxorubicin (1 μg/mL), or a combination of both (a) indicating p53 activation response measured by NLuc activity, and (b) indicating NFκβ transcriptional response measured by hLuz in SKOV3 cell line. Supplementary Figure S8: Effect of Doxorubicin (1 μg/mL), TNFα (10 ng/mL) and combination treatment on p21 (p53 target protein) and BCL2 (NFκβ target protein) expression in A2780 cell line (Treatment for 24 h).
