# Supplementary material for: Triple Reporter Assay: A Non-Overlapping Luciferase Assay for the Measurement of Complex Macromolecular Regulation in Cancer Cells Using a New Mushroom Luciferase–Luciferin Pair
Source: Sensors (Basel). 2023 Aug 22;23(17):7313. doi: 10.3390/s23177313 (PMC10490530; doi:10.3390/s23177313)
Supplement: Supplementary file 1 [file sensors-23-07313-s001.zip › sensors-2554474-supplementary.pdf]

Supplementary information

Triple Reporter Assay: A non-overlapping luciferase assay for measurement of complex macromolecular regulation in cancer cells using a new mushroom luciferase-luciferin pair

Authors –

Aaiyas Mujawar[1,3], Pratham Phadte[2,3], Ksenia A. Palkina[4,5,] Nadezhda M. Markina[4,5], Ameena Mohammad[1], Bhushan L. Thakur[2,3], Karen S. Sarkisyan[4,6], Anastasia V. Balakireva[4,5], Pritha Ray[2,3]*, Ilia Yamplosky[4]* and Abhijit De[1,3]*

Affiliations-

1. Molecular Functional Imaging Lab, Advanced Centre for Treatment, Research and Education in Cancer, Navi Mumbai, INDIA
2. Imaging Cell Signalling and Therapeutics Lab, Advanced Centre for Treatment, Research and Education in Cancer, Navi Mumbai, INDIA
3. Faculty of Life Science, Homi Bhabha National Institute, Mumbai, INDIA
4. Institute of Bioorganic Chemistry (IBCh), Russian Academy of Sciences, Moscow, RUSSIA.
5. Planta LLC, Bolshoi boulevard, 42 str 1, Moscow, Russia
6. Synthetic Biology Group, MRC London Institute of Medical Sciences, London W12 0NN, UK

*Correspondence –

Dr Abhijit De
Molecular Functional Imaging Lab, Advanced Centre for Treatment, Research and Education in Cancer, Navi Mumbai, INDIA
Email: ade@actrec.gov.in

Dr Ilia Yamplosky
Institute of Bioorganic Chemistry (IBCh), Russian Academy of Sciences, Moscow, RUSSIA
Email: ivyamp@gmail.com

Dr Pritha Ray
Imaging Cell Signalling and Therapeutics Lab, Advanced Centre for Treatment, Research and Education in Cancer, Navi Mumbai, INDIA
Email: pray@actrec.gov.in

Other authors' email –

AM: aaiyasmujawar@gmail.com

PP: pratham1122@gmail.com

KP: kpalkina93@gmail.com

NMM: markina.nadya@gmail.com

AMM: ami.cph.ami@gmail.com

BT: bhushan0331@gmail.com

KS: karen.s.sarkisyan@gmail.com

AVB: balakireva.anastacia@gmail.com

Supplementary Table S1. Major chemicals and assay kits used in the study.

**Chemical**	**Catalogue No.**	**Company**
Doxorubicin	D1515	Sigma
Zeocin	R25001	Thermofischer
Ampicillin	695203	Sigma
G418 disulfate salt	G9516	Sigma
BioTrace PVDF membrane	66485	PALL
D-Luciferin	L-8240	Biosynth
Coelenterazine h	C3230	Sigma
Nano-Glo®(Furimazine)	N1110	Promega
TNFα	AF-300-01A	Peprotech
WesternBright ECL kit	K-12045-D20	Advansta
RNA extraction kit	400800	Agilent
Superscript III (cDNA synthesis kit)	11752050	Thermo Fisher
SYBR® Green PCR Master Mix	4309155	Thermo Fisher
Galacto-Light plus system	T1007	Applied biosystems
RIPA lysis buffer	R0278	Sigma
3-OH hispidin	-	Planta LLC
NheI-HF	R3131	New England Biolabs
MluI-HF	R3198	New England Biolabs
DMEM	1210046	GIBCO
RPMI 1640	31800022	GIBCO
Foetal bovine serum	RM10938	HiMedia
Lipofectamine 2000	11668019	Thermo Fischer

Supplementary Table S2. Oligo sequences used in the study

Gene Name	Forward primer	Reverse primer
For hLuz cDNA cloning -		
hLuz	GTTGCTAGCGCCACCATGAGAATCA	ACGACGCGTTTTGGCGTTCTCGACAATCTTGC
For real-time PCR -		
hLuz	AGCAGCAGATCTATCGCCAT	TCTTTTCTCGGGCAGTCTGT
GAPDH	TGCACCACCAACTGCTTAGC	GGCATGGACTGTGGTCATGAG
NFκβIA	CAATGGCCACACGTGTCTAC	CATCAGCCCCACACTTCAAC
For CHIP real-time PCR -		
p53	CGCACAGTACCGAACCCTTAT	AATGCCAACAGAGACTTCAGAAATG
NFκβ	TACGCAGCACCAAGACACTA	GACCTTTTGCTATGCCCTCA

Supplementary Table S3. Antibodies used in the study.

Antibody name	Catalogue No.	Company Name
PIK3CA (p110α) pAb	4255	Cell Signalling Technologies (Danvers, MA, USA)
phospho serine 15 p53 mAb	9286	Cell Signalling Technologies (Danvers, MA, USA)
Total p53 pAb	SC-6243	Santacruz (Texas, USA)
NFκβ (p65) pAb	AB7970	Sigma-Aldrich (USA)
NanoLuc mAb	N7000	Promega (USA)
Luz pAb	NA	In house
α-tubulin mAb	T5168	Sigma-Aldrich (USA)
p21 mAb	12D1	Cell signaling technology
BCL2(N-19) mAb	SC-7382	Santa Cruz biotechnology
HRP-conjugated anti-mouse secondary antibodies mAb	A5316	Sigma-Aldrich (USA)
HRP-conjugated anti-rabbit secondary antibodies mAb	A0545	Sigma-Aldrich (USA)

Supplementary Table S4. Information on Luz and other luciferase used in triple luciferase assay system

**Luciferase**	**Organism**	**Molecular Weight**	**Emission Maxima**	**Substrate**	**Reference**
Firefly Luciferase (FLuc2)	*Photinus pyralis*	62 kD	609 nm	D-luciferin	[1]
NanoLuciferase (NLuc)	*Oplophorus gracilirostris*	19 kD	460 nm	Furimazine	[2]
Fungal Luciferase (Luz)	*Neonothopanus nambi*	27 kD	525 nm	3OH-Hispidin	[3]

References

1. Branchini, B.R.; Southworth, T.L.; Fontaine, D.M.; Kohrt, D.; Florentine, C.M.; Grossel, M.J. A Firefly Luciferase Dual Color Bioluminescence Reporter Assay Using Two Substrates To Simultaneously Monitor Two Gene Expression Events. *Sci. Reports 2018 81* **2018**, *8*, 1–7, doi:10.1038/s41598-018-24278-2.

2. Hall, M.P.; Unch, J.; Binkowski, B.F.; Valley, M.P.; Butler, B.L.; Wood, M.G.; Otto, P.; Zimmerman, K.; Vidugiris, G.; Machleidt, T.; et al. Engineered Luciferase Reporter from a Deep Sea Shrimp Utilizing a Novel Imidazopyrazinone Substrate. *ACS Chem. Biol.* **2012**, *7*, 1848–1857, doi:10.1021/cb3002478.

3. Purtov, K. V.; Petushkov, V.N.; Baranov, M.S.; Mineev, K.S.; Rodionova, N.S.; Kaskova, Z.M.; Tsarkova, A.S.; Petunin, A.I.; Bondar, V.S.; Rodicheva, E.K.; et al. The Chemical Basis of Fungal Bioluminescence. *Angew. Chemie - Int. Ed.* **2015**, *54*, 8124–8128, doi:10.1002/anie.201501779.

(a)

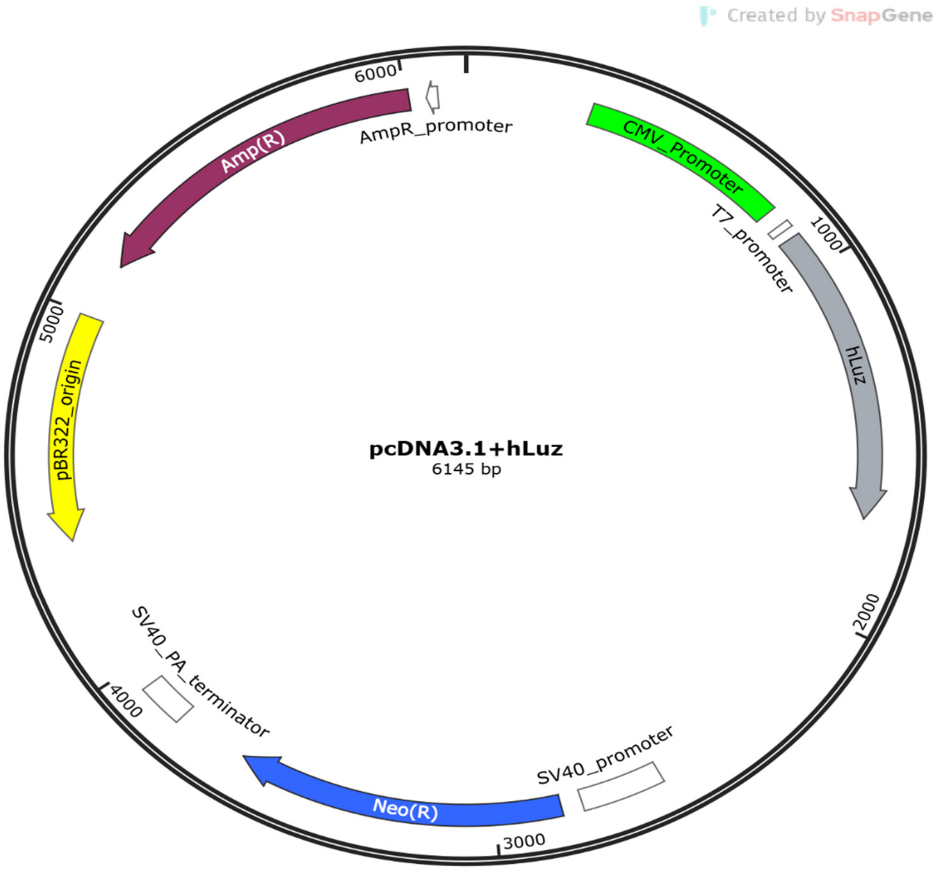

(b)

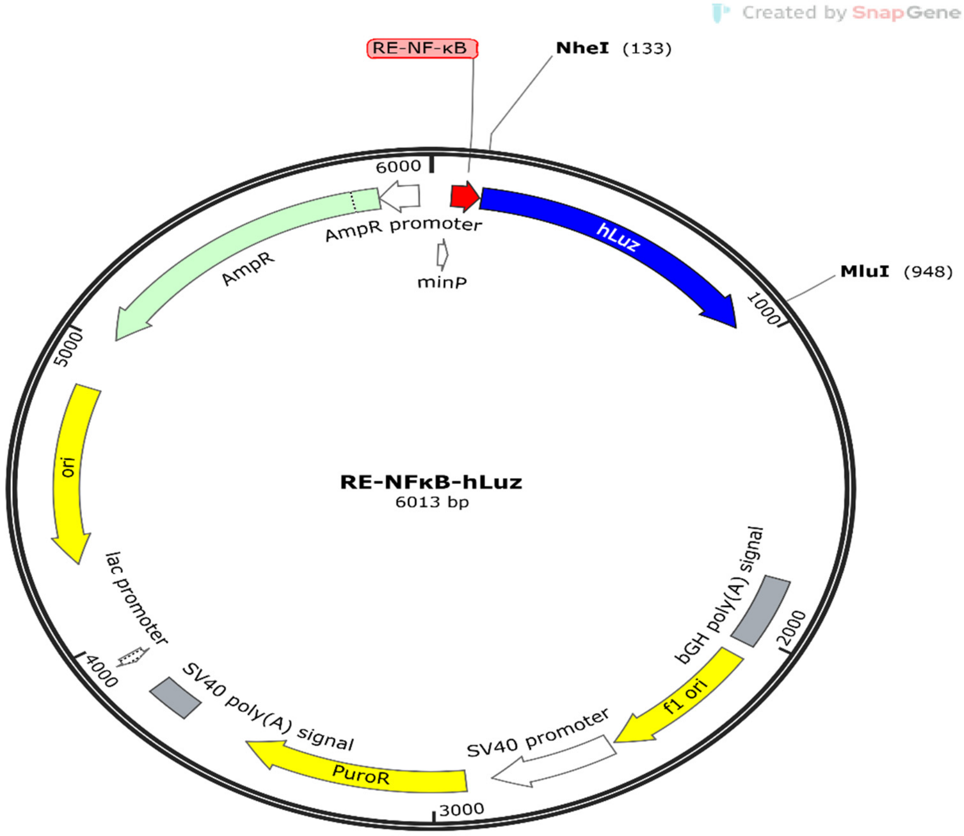

Supplementary Figure S1. Plasmid map of (**a**) pcDNA3.1+hLuz and (**b**) RE-NFκβ-hLuz expression vectors.

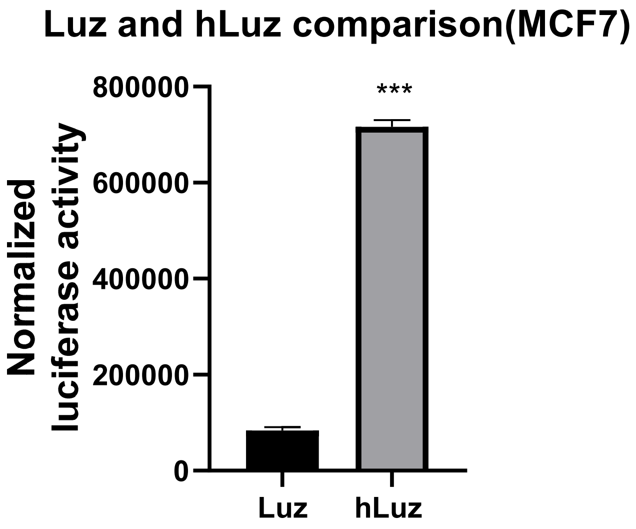

Supplementary Figure S2. Bar graph showing Luz and hLuz activity in MCF7 cell line. Photon output from10000 cells was measured after adding 3-OH hispidin (1mg/ml).

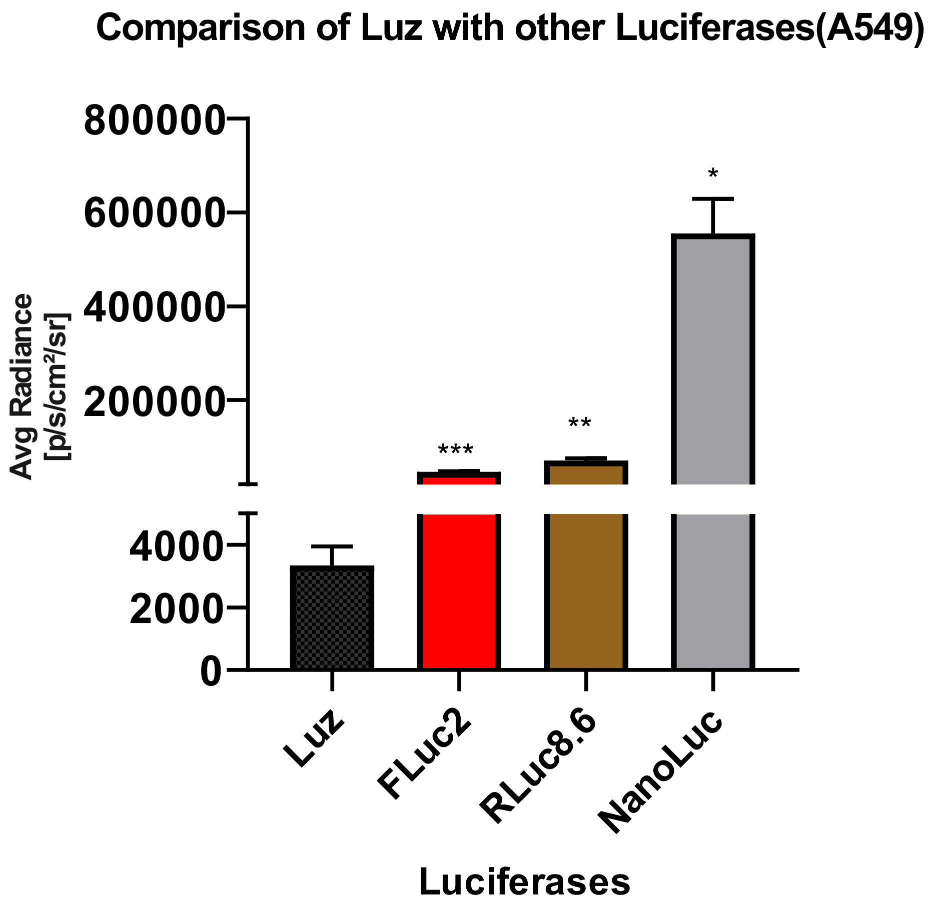

Supplementary Figure S3. Bar graph showing comparative luciferase activity of Luz with FLuc2, RLuc8.6 and NanoLuc in A549 cell line.

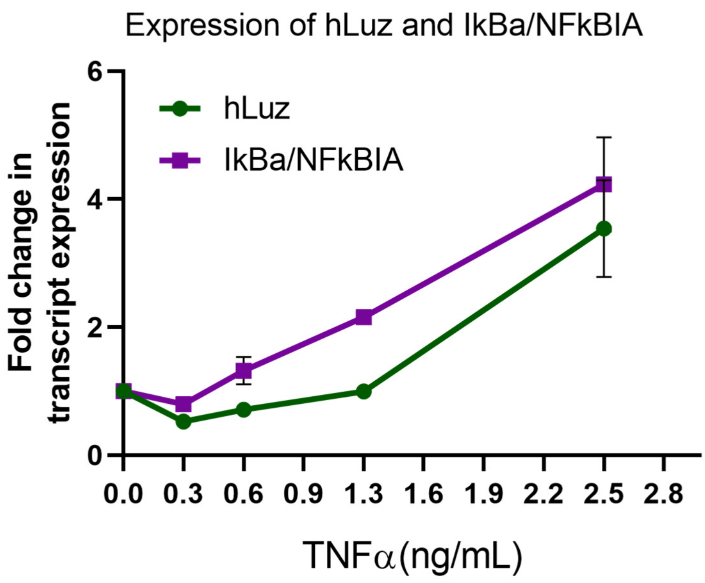

Supplementary Figure S4. Line graph showing fold change of hLuz and NFκβIA transcript expression compared to control in A2780-NhLuz cell line treated with variable TNFα concentration (0.3125,0.625,1.25 and 2.5 ng/mL) for 24h

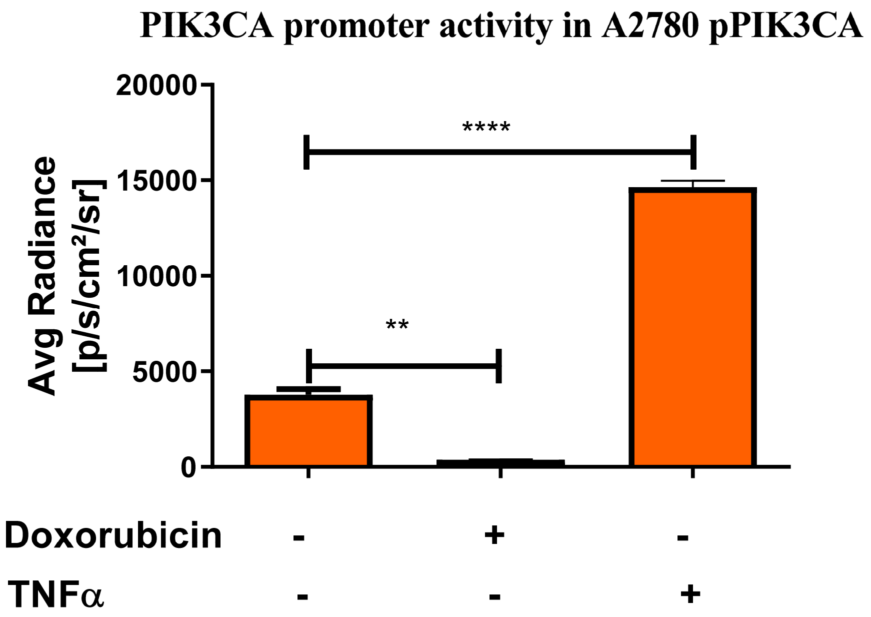

Supplementary Figure S5. Bar graph showing PIK3CA promoter activity in A2780pPIK3CA cell line after treatment of TNFα (10ng/ml) and Doxorubicin(1ug/ml) for 24h.

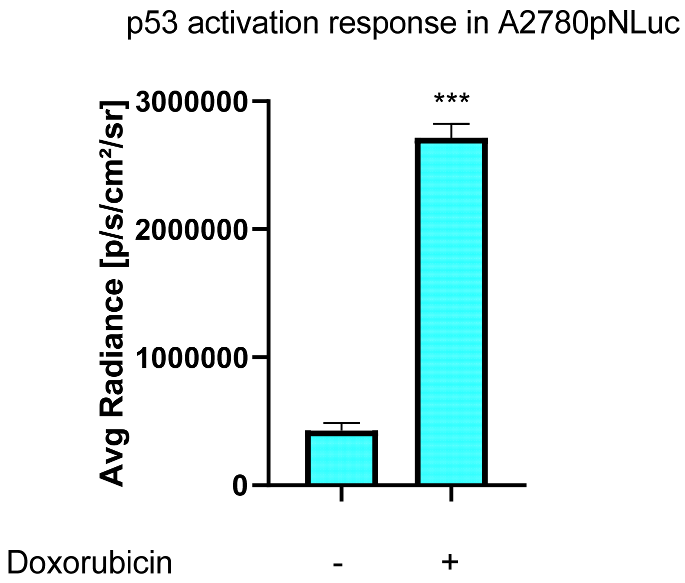

Supplementary Figure S6. Bar graph showing activation of p53 measured by NLuc activity in A2780 cell line over-expressing p53-NLuc fusion protein in presence or absence of Doxorubicin.

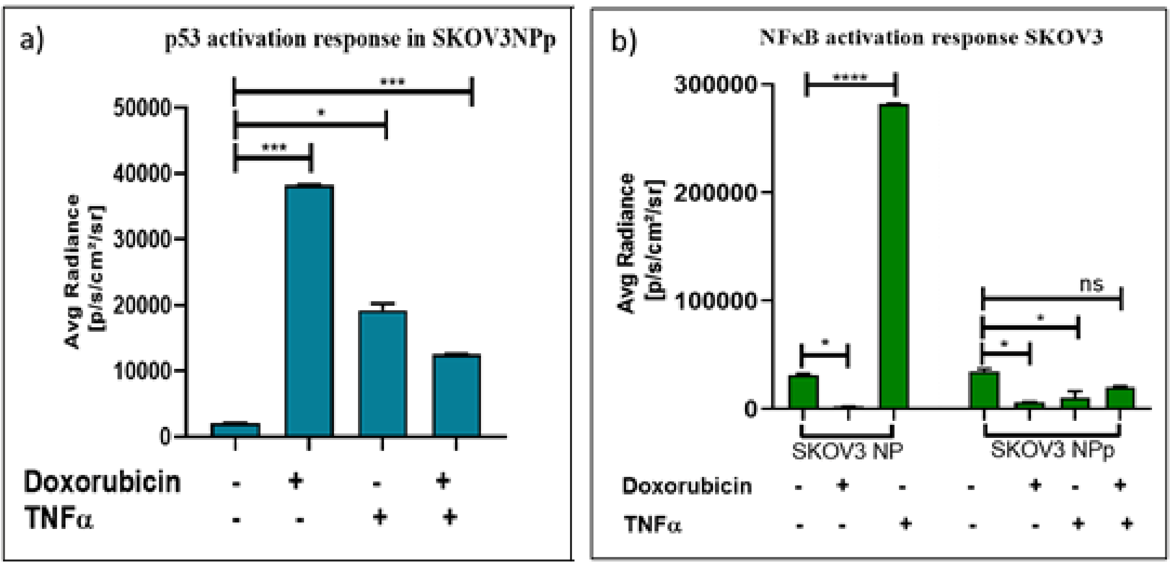

Supplementary Figure S7. Effect of TNFα (10ng/ml), Doxorubicin (1μg/ml), or a combination of both (**a**) indicating p53 activation response measured by NLuc activity, and (**b**) indicating NFκβ transcriptional response measured by hLuz in SKOV3 cell line.

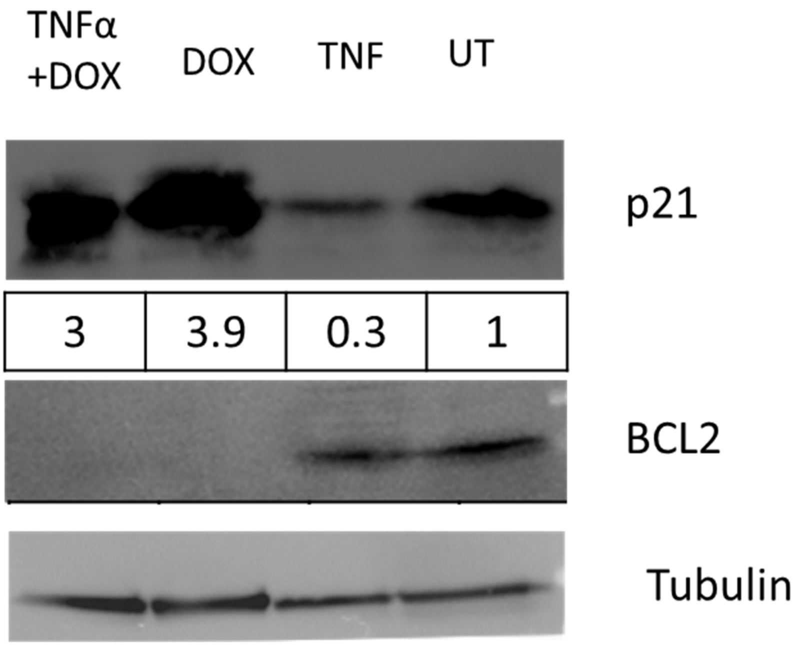

Supplementary Figure S8. Effect of Doxorubicin (1μg/ml), TNFα(10ng/ml) and combination treatment on p21 (p53 target protein) and BCL2 (NFκβ target protein) expression in A2780 cell line (Treatment for 24 hrs). We observe that there is increase in p21 expression after doxorubicin treatment which validates the transcriptional activity of p53. There is slight increase in BCL2 expression with TNFα but drastic decrease with doxorubicin treatment.
